# Balance Ability and Proprioception after Single-Bundle, Single-Bundle Augmentation, and Double-Bundle ACL Reconstruction

**DOI:** 10.1155/2014/342012

**Published:** 2014-12-31

**Authors:** Yubao Ma, Masataka Deie, Daisuke Iwaki, Makoto Asaeda, Naoto Fujita, Nobuo Adachi, Mitsuo Ochi

**Affiliations:** ^1^Department of Musculoskeletal Functional Research and Regeneration, Graduate School of Biomedicine and Health Sciences, Hiroshima University, 1-2-3 Kasumi, Minami-ku, Hiroshima 734-8551, Japan; ^2^Department of Physical Medicine and Rehabilitation, Hiroshima University Hospital, Hiroshima 734-8551, Japan; ^3^Department of Orthopaedic Surgery, Graduate School of Biomedicine & Health Sciences, Hiroshima University, Hiroshima 734-8551, Japan

## Abstract

*Purpose*. The present study sought to determine the influences of single-bundle (SB), single-bundle augmentation (SBA), and double-bundle (DB) reconstructions on balance ability and proprioceptive function. *Methods*. 67 patients who underwent a single- or double-bundle ACL reconstruction or a SBA using multistranded autologous hamstring tendons were included in this study with a 1-year follow-up. Body sway and knee kinesthesia (using the threshold to detect passive motion test (TTDPM)) were measured to indicate balance ability and proprioceptive function, respectively. Additionally, within-subject differences in anterior-posterior stability of the tibia and lower extremity muscle strength were evaluated before and after surgery. *Results*. At 6 and 12 months after surgery, DB reconstruction resulted in better balance and proprioceptive function than SB reconstruction (*P* < 0.05). Although no significant difference was observed in balance ability or proprioceptive function between the SBA and DB reconstructions, knee stability was significantly better with SBA and DB reconstructions than SB reconstruction (*P* < 0.05). No significant differences were found in quadriceps and hamstrings strength among the three reconstruction techniques. *Conclusions*. Our findings consider that joint stability, proprioceptive function, and balance ability were superior with SBA and DB reconstructions compared to SB reconstruction at 6 and 12 months after surgery.

## 1. Introduction

Anterior cruciate ligament (ACL) tears are a common knee injury sustained during sporting and recreational activities. Injury to the ACL causes balance disorder due to mechanical instability of the knee joint [1, 2] as well as proprioceptive dysfunction, both of which compromise further sporting and recreational performance [3]. Mechanoreceptors in the intact ACL contribute to knee joint stability, and damage to these receptors through ACL injuries in turn disturbs neuromuscular control in the knee joint [4–6]. Because improvement of the balance ability is essential for a safe return to most sporting and recreational activities, the proprioceptive dysfunction in the knee joint should be also assessed after ACL reconstruction (ACL-R) surgery. The ACL-R can improve balance disorder due to recovery of mechanical stability and muscle strength [7, 8].

The ACL comprises the anteromedial (AM) and posterolateral (PL) bundles, separated by a septum [9–11]. The two bundles have different roles individually in the important function ACL plays in controlling anterior-posterior translation and rotation of the tibia [12, 13]. The AM bundle is inserted more anteromedially on the tibia and originates more posterosuperiorly on the femur than the PL bundle [14–16], such that the two bundles run parallel in knee extension when the AM bundle tightens and the PL bundle loosens and cross in knee flexion when the AM bundle loosens and the PL bundle tightens [5, 17, 18]. The PL bundle also tightens during internal and external rotation of the knee [5]. In general, there are three types of surgeries to reconstruct the two bundles of the ACL: single-bundle (SB), single-bundle augmentation (SBA), and double-bundle (DB) techniques.

The SB reconstruction uses a graft to replicate the positioning of the intact AM bundle and PL bundle, the SBA reconstruction preserves remnant ACL in addition to using a graft [19, 20], and the DB reconstruction uses two separate grafts to replace the positioning of both the AM and PL bundles [21]. In general, SBA and DB reconstructions restore greater normal knee kinematics than the SB reconstruction. Woo et al. [22] reported that the SB reconstruction cannot completely restore anterior knee stability and is ineffective for retaining rotational stability. Adachi et al. [23] reported superior joint stability with the SBA reconstruction compared to SB techniques, and Li et al. [24] showed that DB reconstructions produced significantly better rotational stability than the SB technique. None of the previous studies showed a significant difference to muscle strength among the surgical techniques [19, 25, 26]. Although differences among the three reconstructions were shown for knee kinematics, effects on balance ability and proprioceptive function are still unclear.

The present study thus sought to reveal the influences of SB, SBA, and DB reconstructions on balance ability and proprioceptive function in patients after ACL surgery. Theoretically, the optimal standard procedure would restore the original ACL anatomical structure comprising the AM and PL bundles [5]. In addition, the DB and SBA reconstructions recover closer-to-normal knee kinematics than the SB reconstruction. Based on the evidence presented, we thus hypothesized that the DB group may show significantly better outcomes in balance ability and proprioception than the SB group, while the augmentation group results would be similar to those for the DB group.

## 2. Materials and Methods

### 2.1. Participants

**Figure 1:**
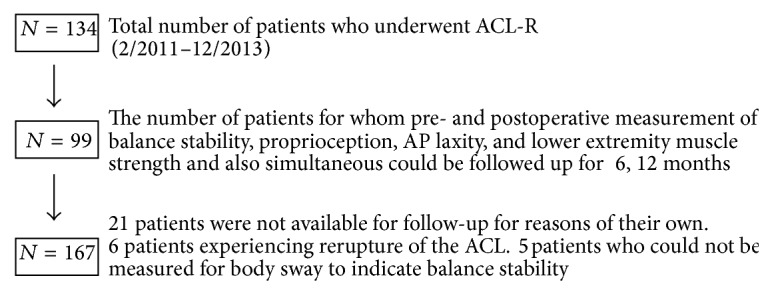
Participants flowchart. Values are presented as the mean ± SD.

The institutional review board at the author's university approved the present study. The participants were informed about the objectives of the study and invited to sign a consent form. From February 2011 to December 2013, 134 patients underwent primary isolated ACL reconstruction or augmentation using multistranded autologous hamstring tendons at the author's university hospital. In the present study, body sway, knee kinesthesia, anterior-posterior knee laxity, and lower extremity muscle strength were assessed in 99 participants before and after surgery. 35 patients were excluded who have other lower extremity diseases, meniscus injury, complex ligament damage, and so on. The patients who experienced rerupture of the ACL (*n* = 6), could not stand on one leg (*n* = 5), and/or were not available for follow-up at 12 months after the ACL reconstruction (*n* = 21) were excluded. Finally, sixty-seven patients (32 male and 35 female) were included in this study (Figure 1). Basic information of these patients is detailed in Table 1.

### 2.2. Surgical Technique

The senior orthopedic specialist at our hospital performed all surgeries. Routine arthroscopic inspection was performed through lateral and medial infrapatellar portals using a 30-oblique arthroscope with the knee flexed at 90°. The decision on surgical technique was based on the following criteria: patients with a complete rupture of the ACL with no ligamentous tissue remaining in the anatomic femoral attachment and those who underwent either a lower femoral tunnel-placed SB ACL reconstruction (anatomical central SB ACL-R) or an anatomical DB ACL-R. SB or DB technique was indicated based on several factors, such as the size and length of the semitendinosus tendon and width of the intercondylar notch. An ACL augmentation was chosen in the case of partial ACL rupture [20, 23, 27–29].

In the anatomical central SB ACL-R procedure the tibial tunnel was created at the point 2 mm anterior and 2 mm medial to the center of its attachment from the medial aspect of the proximal tibia, and the femoral tunnel was centrally located between the femoral attachments of the AM and PL bundles. An autologous quadrupled semitendinosus tendon was used to make a graft.

For an anatomical DB ACL-R, both tibial and femoral insertions of the AM and PL bundles were created at the center of each attachment of the AM and PL bundles, and two autologous doubled semitendinosus tendons were used to make the grafts. Details of this procedure were described in a previous report [28].

Several previous studies described the SBA ACL-R procedure [20, 23, 29]. It should be noted that this ACL augmentation procedure is a central SB ACL augmentation to preserve the ACL remnants as opposed to a PL or AM bundle reconstruction. The ACL graft and the graft fixation method were the same as for the SB ACL-R. All patients followed the same postoperative rehabilitation program regardless of the ACL procedure.

### 2.3. Balance Ability

Body sway was measured to indicate balance ability, using the equilibrium function meter G-620 (Anima, Tokyo, Japan). Briefly, subjects maintained 20 seconds of single-leg standing with eyes closed on the equilibrium function meter. To make sure the measurement does not fail, twenty seconds was chosen. Body posture at the time of measurement was applied to the opposite side shoulder with both hands, and subjects were instructed to mildly elevate the foot with the standing leg mildly flexed. After a lapse of 5 seconds, we recorded the total length of body sway with 20 seconds.

### 2.4. Proprioceptive Function of the Knee Joint

**Figure 2:**
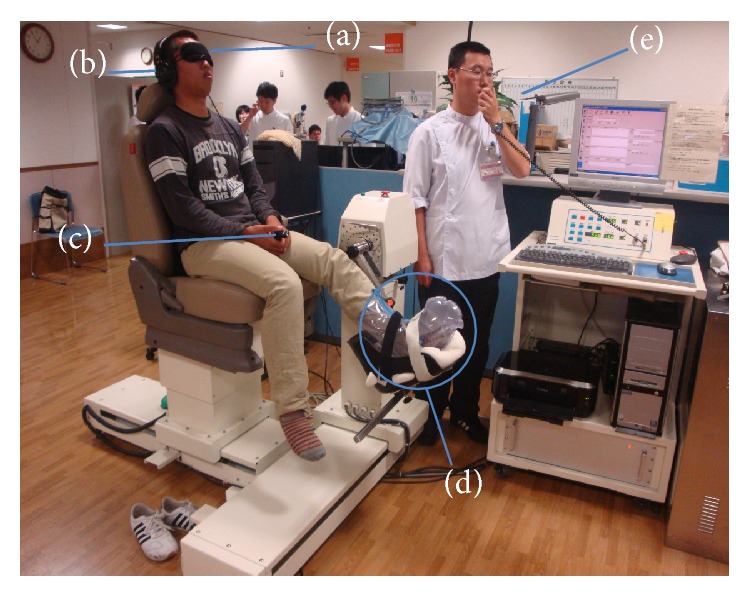
Measurement of proprioceptive function of the knee. (a) Eye mask, (b) headphone, (c) stop switch, (d) air splint, and (e) microphone.

Kinesthesia was measured to indicate proprioceptive function and measured using a self-designed proprioception testing apparatus (Sensor Ouyou, Hiroshima, Japan; Figure 2), which was developed as described previously [30]. We assessed knee joint proprioception three times: before the operation and at 6 and 12 months of follow-up by measuring the subjects' threshold to detect passive motion (TTDPM), represented by the reaction time from the movable shaft starting movement to the point at which patients pressed the stop switch. The data were obtained from the injured side at each measurement. According to the previous study [31], we set up the starting positions at 45° flexion of the knee joint, and the movable shaft was shifted gradually by 0.2°/s toward either the extension or flexion direction at random.

As preparation for the measurement, the patients were seated in a neutral angle of lumbar flexion with the popliteal fossa situated approximately 5 cm from the edge of the seat. The test limb was put into an air splint that applies pneumatic compression to eliminate any cutaneous stimulation and to minimize neural input from mechanoreceptors in the foot and ankle. All subjects were blindfolded with eye masks to eliminate visual clues to knee position. To eliminate auditory clues to the start of knee flexion, participants wore a set of headphones and listened to “white noise” during the test. After the preparation, the axis of rotation of the limb being tested and the axis of the movable shaft were aligned. The movable shaft was connected to a motor-driven rotational transducer interfaced with a computer to measure reaction time to the passive motion. A hand-held switch enabled each subject to stop the movement when they perceived it as joint motion. During the test, the subjects were instructed via a microphone attached to the testing device. Patients were instructed to press the switch when they felt their lower limbs moving towards either flexion or extension from starting position. The times that patients took to press the button were recorded and analyzed statistically.

### 2.5. Knee Joint Stability

In the present study, AP laxity of the knee was measured pre- and postoperatively by Knee Lax3 (Monitored Rehab Systems, Netherlands) with 133N at the knee angle 20°. We measured the injured and noninjured sides at least three times each with the largest displacement, and side-to-side difference (SSD) was calculated by subtracting the noninjured side value from that of the injured side.

### 2.6. Quadriceps and Hamstrings Strength

Measurements of concentric isokinetic strength of the knee muscles were performed with a Biodex Multi-Joint System 3 isokinetic dynamometer (Biodex Medical Systems, Inc., Shirley, NY, USA) with Biodex Advantage software, version 4.5 (Biodex Medical Systems, Inc.), using the standard Biodex knee unit attachment. Subjects were placed in an upright position with 90° of hip flexion on the dynamometer chair and were secured with straps across the chest, pelvis, thigh, and ankle. The resistance pad was placed as distally as possible on the tibia while still allowing full dorsiflexion at the ankle. The center of motion of the lever arm was aligned as accurately as possible with the lateral epicondyle flexion-extension axis of the knee joint. The range of motion of the knee joint was set at 0–90°. The subjects gripped the handles of the bench to stabilize their body during the test. Standardized oral instructions and encouragement were given. The subjects were allowed twice trial tests to familiarize themselves with the equipment and the test procedure before five maximal reciprocal concentric isokinetic knee extensions and flexions at an angular velocity of 60°/s were made. The maximum torque value was normalized to body weight which was used in the analysis.

### 2.7. Statistical Analyses

The data were expressed as mean ± SD. Statistical analyses were performed using SPSS. Significant differences in patient values among the three reconstructions were analyzed using one-way ANOVA followed by the Tukey-HSD post hoc test. For the values of body sway, TTDPM, anterior-posterior knee laxity, and quadriceps and hamstrings strength, significant differences among the three reconstructions or within reconstructions over time were analyzed using a two-way ANOVA followed by using the two-factor (treatment × time) post hoc test. The statistically significant level was set at *P* < 0.05.

## 3. Results

### 3.1. Body Sway

**Figure 3:**
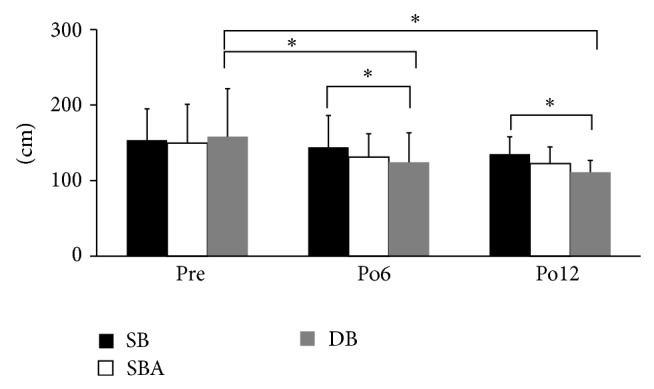
Total length of center of pressure. The value in the DB reconstruction was significantly shorter than that in the SB reconstruction at 6 and 12 months after ACL-R (^*^ *P* < 0.05). In the DB reconstruction, the values at 6 and 12 months after ACL-R were significantly shorter than the preoperative value (^*^ *P* < 0.05).

The total lengths of center of pressure were significantly shorter in the DB reconstruction than in the SB reconstruction at 6 (DB, 124.3 ± 39.0 cm; SB, 144.0 ± 42.2 cm) and 12 (DB, 111.0 ± 15.5 cm; SB, 134.9 ± 23.0 cm) months after ACL-R (*P* < 0.05, Figure 3). The values in the SBA and DB reconstructions were almost identical, and there were no significant differences at 6 and 12 months after ACL-R and preoperatively. The values in the DB reconstruction were significantly smaller at 6 (124.3 ± 39.0 cm) and 12 (111.0 ± 15.5 cm) months after ACL-R than the preoperative (158.1 ± 63.4 cm) value (*P* < 0.05, Figure 3).

### 3.2. TTDPM

**Figure 4:**
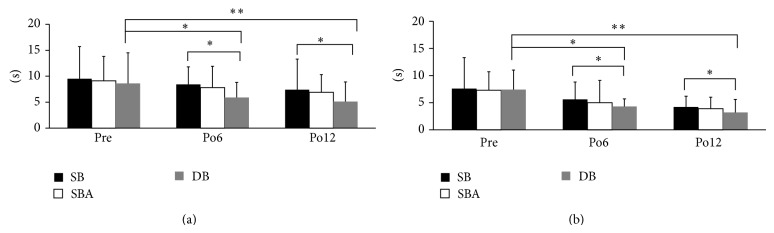
Proprioceptive reaction time for the knee in extension (a) and flexion (b). The time in the DB reconstruction was significantly shorter than that in the SB reconstruction at 6 and 12 months after ACL-R (^*^ *P* < 0.05). In the DB reconstruction, the times at 6 and 12 months after ACL-R were significantly shorter than the preoperative value (*P* < 0.05, ^**^ *P* < 0.01).

The reaction times of knee flexion and extension were significantly more following the DB reconstruction than the SB reconstruction at 6 and 12 months after ACL-R (*P* < 0.05, Figure 4). The values of flexion were 4.3 ± 1.4 s and 3.2 ± 2.4 s with DB, while the SB was 5.6 ± 3.2 s and 4.2 ± 2.0 s at postoperative 6 and 12 months; the extension was 5.9 ± 2.9 s and 5.1 ± 3.8 s with DB, while the SB was 8.4 ± 3.4 s and 7.4 ± 5.9 s after reconstruction at 6 and 12 months. No significant differences were found between the SBA and DB reconstructions at 6 and 12 months after ACL-R. The reaction times in the DB reconstruction were significantly lower at 6 and 12 months after ACL-R than the preoperative value (*P* < 0.05, *P* < 0.01, Figure 4). The values of flexion were 7.4 ± 3.6 s; the extension was 8.6 ± 5.9 s preoperatively.

### 3.3. Anterior-Posterior Stability

**Figure 5:**
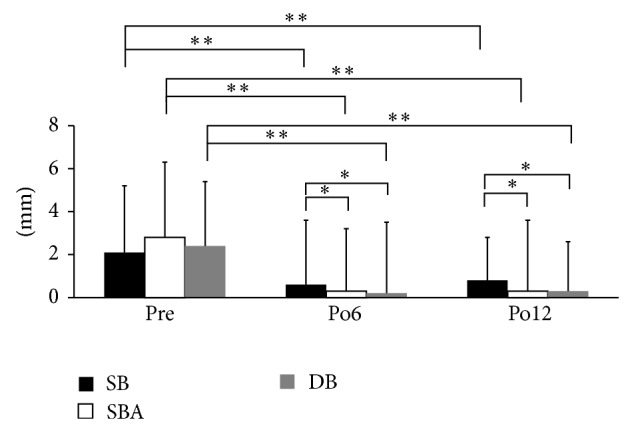
Knee stability measured by KT-2000. The values with the SBA and DB reconstruction were significantly lower than that with the SB reconstruction at 6 and 12 months after ACL-R (^*^ *P* < 0.05). In all reconstructions, the values at 6 and 12 months after ACL-R were significantly lower than the preoperative value (*P* < 0.05, ^**^ *P* < 0.01).

The values of anterior-posterior knee stability were significantly smaller with the SBA and DB reconstructions compared to the SB reconstructions at 6 and 12 months after ACL-R (*P* < 0.05, Figure 5). The SSD of DB was 0.2 ± 3.3 mm and 0.3 ± 2.3 mm, and SBA was 0.3 ± 2.9 mm and 0.3 ± 3.3 mm, while SB group was 0.6 ± 3.0 mm and 0.8 ± 2.0 mm at postoperative 6 and 12 months. There were no significant differences between the SBA and DB reconstructions at 6 and 12 months after ACL-R. The values in all reconstructions were significantly smaller at 6 and 12 months after ACL-R than the corresponding preoperative values (*P* < 0.01, Figure 5). The values of DB were 2.4 ± 3.0 mm, SBA was 2.8 ± 3.5 mm, and SB was 2.1 ± 3.1 mm preoperatively.

### 3.4. Quadriceps and Hamstrings Strength

**Figure 6:**
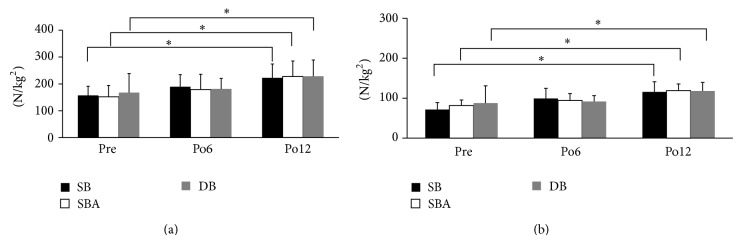
Preoperative and postoperative muscle strength for the knee in extension (a) and flexion (b). The values at 12 months after ACL-R were significantly larger than the preoperative value with each reconstruction (^*^ *P* < 0.05).

There were no significant differences in strength of the knee extensor and flexor muscles among the three reconstruction techniques at 6 and 12 months after ACL-R (Figure 6). The values for all reconstructions were significantly larger at 12 months after ACL-R than the preoperative values (*P* < 0.05, Figure 6).

## 4. Discussion

The present study revealed that balance ability as measured by body sway and proprioceptive function based on TTDPM was better in patients who underwent a DB reconstruction than in those undergoing an SB reconstruction at 6 and 12 months after ACL-R. Additionally, mechanical joint stability measured by anterior-posterior stability was better with the DB and SBA reconstructions than the SB reconstruction at 6 and 12 months after ACL-R. However, there was similar quadriceps and hamstrings strength among the three types of reconstruction compared to preoperative values. These results suggest that early improvement of proprioceptive function and joint stability enhances balance ability in patients treated by DB or SBA reconstructions, supporting our initial hypothesis.

As shown here, many studies have demonstrated superior anterior stability following a DB reconstruction compared with SB reconstruction techniques [24, 32–34]. Adachi et al. [23] also compared the anterior stability between the SBA and SB reconstructions and showed the former to be superior. In the SB reconstructions, the replacement graft was attached only at the insertion point between the AM and PL bundles [19, 35]. On the other hand, in the DB reconstruction, the grafts were attached at the insertions of the AM and PL bundles, respectively [19, 28, 35]. The PL bundle is the primary restraint to tibial anterior translation and rotation in knee extension [5, 12, 13]. Thus, because the SBA and DB reconstruction are inserted at the PL bundle, the anterior stability must be superior compared with the SB reconstruction without the insertion of the PL bundle.

Proprioception has been defined as the afferent information arising from the internal peripheral area of the body and contributing to postural control, joint stability, and specific conscious sensations [36]. An ACL injury will reduce knee proprioception, possibly through disruption of mechanoreceptors within the ligament [37, 38]. Accordingly, an ACL-R can improve proprioception due to recovery of mechanical stability. In the present study, mechanical joint stability measured by anterior-posterior stability was better with the DB and SBA reconstructions than the SB reconstruction at 6 and 12 months after ACL-R, indicating that better knee proprioception can be achieved with the DB and SBA reconstructions compared to the SB reconstruction.

Moreover, the AM and PL bundles tighten in different knee positions [5], and because ACL proprioception is excited by the stretch stimulation [39–41], the SBA and DB reconstructions with two insertions must restore higher sensitivity compared with the SB reconstruction. Proposed features of ACL injury are decreased postural control, as defined by higher amplitudes of center of pressure movements, and decreased proprioception, as defined by higher TTDPM values [42, 43]. Additionally, impairment of proprioception may lead to increased postural sway and, potentially, the loss of balance [44]. Finally, Lee et al. [45] reported that poorer proprioceptive function results in poorer balance. Therefore, the superiorities in joint stability and proprioceptive function in the SBA and DB reconstructions compared to the SB technique are thought to underpin the enhanced balance ability.

Although postoperative balance ability was better in the SBA and DB reconstructions than in the SB reconstruction, there were no significant differences in postoperative muscle strength among the reconstructions. Graft harvesting issues are thought to attenuate strength of the knee flexor after ACL-R [46–48]; however, most of the muscle weakness induced by graft harvest was noted only in the early postoperative period and improved during the several postoperative months [49]. In contrast, some studies reported no significant difference in recovery of muscle strength between the SB and DB reconstructions at 6 months after ACL-R [19, 25, 26]. However, because these and the present study measured the strengths of knee extensor and flexor muscles only at 6 months after ACL-R, differences among the reconstructions might not yet be apparent. Furthermore, the muscle strength is influenced by age, gender, body size, and waiting period for surgery, and patients in the present study were matched for such factors that affect muscle strength, lessening any observable difference among techniques. In addition, Lee et al. [45] reported that lower extremity muscle strength does not influence balance ability in the ACL-injured leg, and poor correlation has been shown between muscle strength and body sway [50]. Therefore, it is reasonable to assume that muscle strength has a low impact on recovery of balance ability after ACL-R.

The limitation of the present study is that we could not strictly determine the amount and attachment of the remnant ligament tissue in SBA reconstructions. Our surgical method followed the technical note shown by Ochi et al. [20], and whether the remnant was preserved was decided based on the macroscopic anatomy. In the present study, the results of the SBA and DB reconstructions were almost identical. However, we need further data to focus in more detail on the difference between SBA and DB reconstructions.

## 5. Conclusion

Joint stability, proprioceptive function, and balance ability were superior in patients following the SBA and DB reconstructions compared to those after the SB reconstructions at 6- and 12-month follow-up. Additionally, the DB reconstructions achieved an early recovery of balance ability and proprioceptive function. These results suggested that it is possible ACL-R using the DB or SBA techniques is safe and effective in enhancing early return to sporting and recreational activities due to improved balance ability and proprioceptive function.

## Figures and Tables

**Table 1:** Basic information about study participants (at surgery).

	SB	SBA	DB	*P*
	(*n* = 20)	(*n* = 21)	(*n* = 26)	
Male/female	10/10	11/10	11/15	
Age (y)	25.2 ± 1.3	29.5 ± 1.3	26.7 ± 1.4	N.S.
Body height (cm)	164.2 ± 1.1	167. 3 ± 0.8	167.8 ± 0.8	N.S.
Body weight (kg)	62.3 ± 2.7	65.4 ± 1.2	66.9 ± 2.1	N.S.
BMI (kg/m^2^)	23.2 ± 2.2	23.4 ± 1.9	23.7 ± 3.3	N.S.
Injury-pre-op time (month)	4.7 ± 3.3	3.9 ± 2.7	5.2 ± 2.1	N.S.

Values are presented as mean ± SD. SB, single-bundle reconstruction; SBA, single-bundle augmentation; DB, double-bundle reconstruction; N.S., not significant.
